# Splice variants of zinc finger protein 695 mRNA associated to ovarian cancer

**DOI:** 10.1186/1757-2215-6-61

**Published:** 2013-09-05

**Authors:** Sergio Juárez-Méndez, Alejandro Zentella-Dehesa, Vanessa Villegas-Ruíz, Oscar Alberto Pérez-González, Mauricio Salcedo, Ricardo López-Romero, Edgar Román-Basaure, Minerva Lazos-Ochoa, Víctor Edén Montes de Oca-Fuentes, Guelaguetza Vázquez-Ortiz, José Moreno

**Affiliations:** 1 Experimental Oncology Laboratory, Research Department, National Institute of Pediatrics, Mexico, D. F, Mexico; 2 Mexican Faculty of Medicine, Universidad La Salle, Mexico City, Mexico; 3 Genomic Medicine and Environmental Toxicology Department, Biomedical Research Institute, UNAM, Mexico, D. F, Mexico; 4 Unit of Biochemistry, National Institute of Medical Sciences and Nutrition “Salvador Zubirán”, Mexico City, Mexico; 5 Genomic Oncology Laboratory, Medical Research Unit in Oncologic Diseases, Oncology Hospital, National Medical Center Siglo XXI, IMSS, Mexico City, Mexico; 6 Faculty of Medicine, UNAM, General Hospital of México O. D, Mexico, D. F, Mexico; 7 Department of Oncology, General Hospital of México O. D, Mexico, D. F, Mexico; 8 Mammalian Genetics Section, NIDDK, National Institutes of Health, 10/9 N104, 10 Center Drive, Bethesda, MD 20892, USA

**Keywords:** Ovarian cancer, Alternative mRNA splicing, ZNF695

## Abstract

**Background:**

Studies of alternative mRNA splicing (AS) in health and disease have yet to yield the complete picture of protein diversity and its role in physiology and pathology. Some forms of cancer appear to be associated to certain alternative mRNA splice variants, but their role in the cancer development and outcome is unclear.

**Methods:**

We examined AS profiles by means of whole genome exon expression microarrays (Affymetrix GeneChip 1.0) in ovarian tumors and ovarian cancer-derived cell lines, compared to healthy ovarian tissue. Alternatively spliced genes expressed predominantly in ovarian tumors and cell lines were confirmed by RT-PCR.

**Results:**

Among several significantly overexpressed AS genes in malignant ovarian tumors and ovarian cancer cell lines, the most significant one was that of the zinc finger protein ZNF695, with two previously unknown mRNA splice variants identified in ovarian tumors and cell lines. The identity of ZNF695 AS variants was confirmed by cloning and sequencing of the amplicons obtained from ovarian cancer tissue and cell lines.

**Conclusions:**

Alternative ZNF695 mRNA splicing could be a marker of ovarian cancer with possible implications on its pathogenesis.

## Background

Ovarian Cancer (OC) is the sixth most prevalent form of cancer worldwide, which has a high mortality rate because at the time of diagnosis nearly 70% of cases are at an advanced stage, leading to a 5 year survival below 30% [1]. The classification of OC depends on its cellular origin, with approximately two-thirds belonging to the epithelial serous type [2]. Similar to other types of cancer, OC is characterized by changes in gene expression profiles [3-6], including under and overexpression, or even *de novo* gene expression [7,8].

Alternative splicing (AS) provides a critical and flexible layer of regulation, intervening in many biological processes, such as the diversity of proteins.

AS has major impact on the cell phenotype as a single pre-mRNA spliced in different ways can give rise to different mature mRNA transcripts (variants) that are translated onto distinct proteins varying in functions [9-11]. Apparently, over 90% of human genes have two or more splice variants [9,10], greatly increasing the complexity of both the transcriptome and the proteome [12,13]. Therefore, AS could play an important role in gene regulation both in health and disease. In cancer, AS could affect the cellular processes related to tumor progression, including inhibition of apoptosis, tumor invasiveness, metastasis and angiogenesis [14].

Among the genes with well established AS patterns whose derived alternative proteins affect tumor cell behavior is the SRPK1 kinase that in breast, colonic and pancreatic carcinomas phosphorylates the splicing factor SF2/ASF, allowing import to the nucleus, where it modulates AS of multiple target mRNAs, such as BIN1, S6K1, MNK2, contributing to tumor progression [15,16]. Additional cancer types with apparent alterations of alternative splicing, include: gastric, colon and bladder carcinomas [17], hepatocarcinoma [18], prostatic cancer [19,20], multiple myeloma [21], breast cancer, and OC [22], where most cancer-associated transcript variants belong to genes related to processes such as cellular transformation [23,24], adhesion, proliferation, migration and invasion [25-28]. In OC, a new, previously unknown, variant of p53 mRNA transcript variant (p53δ) was identified [29] whereas variants of the NR4A1, a nuclear receptor involved in steroidogenesis, and MRRF, a mitochondrial protein, were identified in prostatic cancer [20]. Although the extent and pathophysiological meaning of this has yet to be established, there is little doubt that the study of alternative splicing can lead to a better understanding of the mechanisms of cancer development, and to the identification of new biomarkers for the diagnosis, epidemiological studies of prevalence, prognosis, and therapeutic responses.

The aim of the present study was to identify the whole genome profile of alternatively spliced mRNA in ovarian cancer and cell lines by high-density microarrays. Among the spectrum of several ovarian cancer-associated alternatively spliced genes, one mRNA, coding for ZNF695, a zinc finger protein, had the most significantly overexpression in OC with two prominent splice variants that were not present in normal ovarian tissue. These variants were cloned and sequenced. Here we describe some of the characteristics of ZNF695 mRNA splicing variants associated to ovarian cancer.

## Methods

### Data set and specimens

All investigations were performed in accordance with the Declaration of Helsinki with approval by the Central Research Committee of the Mexican Institute of Social Security and The Ethics Committee of Centro Médico Siglo XXI, Mexican Institute of Social Security. After informed consent was obtained, normal ovarian tissue (HOT), borderline ovarian tumor (BOT), malignant epithelial ovarian tumors stages III and IV (MOT) tissues were collected by the clinical partners at the Oncology Hospital, National Medical Center Siglo XXI, IMSS, and at the General Hospital of Mexico SSA (Secretaría de Salud) from patients with diagnosed ovarian cancer, or healthy ovarian tissue from patients who underwent abdominal surgery for hysterectomy due to uterine myomatosis with no evidence of ovarian pathology. Routinely, during this type of procedure, in patients over 45 years old both ovaries are removed, and only one in patients under 45.

Cancer and corresponding normal tissue specimens were cut into three fragments and snapped frozen in liquid nitrogen, one of which was stored in RNA Latter^R^ (Qiagen, Valencia, CA, USA) at −70°C for a maximum of two months until RNA was purified, and the other two remaining fragments were formalin-fixed, paraffin-embedded, sliced, mounted on slides, and stained with HE. Only tissue samples with >80% tumor cells or normal epithelial cells (MOT or HOT, respectively), according to the histopathological examination were included for analysis.

Moreover, we also included for study ovarian cancer cell lines (OCL) NIH: OVCAR-3 [30], SK-OV-3 [31], TOV-112D [32] and TOV-21G [32], kindly provided by Dr. Laura Díaz-Cueto, Research Unit on Reproductive Medicine, Instituto Mexicano del Seguro Social (IMSS).

Samples were disrupted using a TissueLyser™ system (Qiagen, Valencia, CA, USA) for 60s at 30 Hz. Total RNA was obtained with RNeasy Mini Kit (Qiagen, Valencia, CA, USA) and total RNA concentration was quantified using a NanoDrop ND-1000 spectrophotometer and RNA quality was visualized and measured on an Agilent RNA 6000 Nano Assays in an Agilent 2100 Bioanalyzer (Agilent Technologies, Santa Clara, CA, USA).

#### Microarray GeneChip 1.0 assay

The microarray used for these studies was Affymetrix GeneChip 1.0, which contains over 750,000 probe sets representing all exons of ~28,800 annotated genes. Sample amplification and preparation for microarray hybridization was performed according to Affymetrix specifications (http://media.affymetrix.com/support/downloads/manuals/wt_expressionkit_manual.pdf). In brief, 100 ng total RNA was reverse transcribed to cDNA, amplified by *in vitro* transcription and reverse transcribed to cDNA again. Fragments between 40 and 70 bp were generated enzymatically, labelled and hybridized onto the microarray chips in an Affymetrix hybridization oven at 60 rpms, 45°C for 17 hours. Chips were washed according to the established protocols (Affymetrix, Santa Clara, CA, USA) with GeneChip fluidics station 450, and finally they were scanned with an Affymetrix 7G GeneChip scanner. The raw data (CEL files) will be deposited in Gene Expression Omnibus (GEO).

### Data analysis

Microarray analysis was achieved by means of CEL files of the Partek Genomics Suite 6.5 v software (Partek Incorporated, Saint Louis, MO). Probe sets were summarized by means of Median Polish and normalized by quantiles with no probe sets excluded from analysis. Background noise correction was achieved by means Robust Multi-chip Average (RMA) and data were log2 transformed. Data grouping and categorization was achieved by principal component analysis (PCA). Differentially expressed exons were detected by means of Alternative Splicing ANOVA with the healthy control samples as the baseline. Moreover, BOT, MOT and OCL were also examined against HOT by the Geometric least squares means model. Hierarchical clustering was based on the dissimilarity of samples (Euclidian method) by means of average linkage.

### Reverse transcription PCR

For linear cDNA synthesis, 1 μg total RNA was predigested with 1 U DNAse, 1 × DNAse buffer, 5 mM EDTA, after which it was incubated at 37°C for 30 min and at 65°C for an additional 10 min. Thereafter, samples were placed in master mix containing: 40 U Ribolock RNAse inhibitor, 0.2 μg random hexamer primers, 20 mM dNTP’s mix, 40 U M-Mulv reverse transcriptase (RT), and 1 × M-Mulv RT Buffer (Thermo Scientific).

Conditions for endpoint PCR amplification were: 5′ CGAATGAGAGCTGGCAAAGGCAAA 3′ Fwd., 5′ ACGCCAAGTGCCGTACAATTCATC 3′ Rev. primers (housekeeping gene RPL4) 7.5 mM, 1 × Taq buffer, 2 mM MgCl_2_, 0.4 mM dNTP’s, 1.25 U Taq Pol, 2 ul cDNA; whereas ZNF695 was amplified with primers: 5′ GCCTTTGTCTCCTTGCGGC 3′ Fwd. 5′ GGCTGTCTTCTCTGTGTTCACGTT 3′ Rev. 12.5 mM, 1 × Taq buffer, 3 mM MgCl_2_, 0.4 mM dNTP’s, 1.25 U Taq Pol, 2 ul cDNA. In both cases mix reactions were initially incubated at 95°C for 5 min, and then were run for 40 cycles at 94°C 45 s, 59°C 45 s, 72°C 60 s; and finally at 72°C for 5 min.

### PCR product purification and cloning

PCR products were separated by electrophoresis (2.5% agarose gels) and extracted by means of Gel extraction kit^TM^ (Qiagen, Valencia, CA, USA). The extracted products were ligated into pGem-T Easy Vector™ (Promega, Madinson, WI) by incubating overnight in 1.5 mL Eppendorf tubes with 2 × Rapid Ligation Buffer (T4 ligase), pGEM-T Easy Vector, PCR product and T4 DNA ligase at 4°C.

Recombinant plasmid DNA was purified with Wizard Plus Miniprep DNA Purification System™ (Promega) and selected clones were sequenced with M13 oligonucleotide and BigDye Terminator 3.1 cycle sequencing kit (Applied Biosystems), and sequenced in an Applied Biosystems Abi Prism 3130 genetic analyzer automated sequencer. Subsequently, the PCR amplicon sequences were assembled and checked against the transcript sequences annotated in the NCBI nucleotide database.

## Results

### Expression microarray assays

A total of 14 samples with an RNA integrity number (RIN) ≥ 8 were hybridized in GeneChip 1.0 microarrays according to the MIAME guidelines. Histopathological classification of tissues was as follows: healthy ovarian tissue (HOT) n = 4, benign ovarian tumors (BOT) n = 2, (malignant) serous epithelial ovarian tumors in stages III and IV (MOT) n = 4, and ovarian cell lines (OCL) n = 4. As a prerequisite, healthy tissue had to be free of any visible alteration, whereas all tumor tissues, benign or malignant, selected for study contained at least 90% tumor cells.

**Figure 1:**
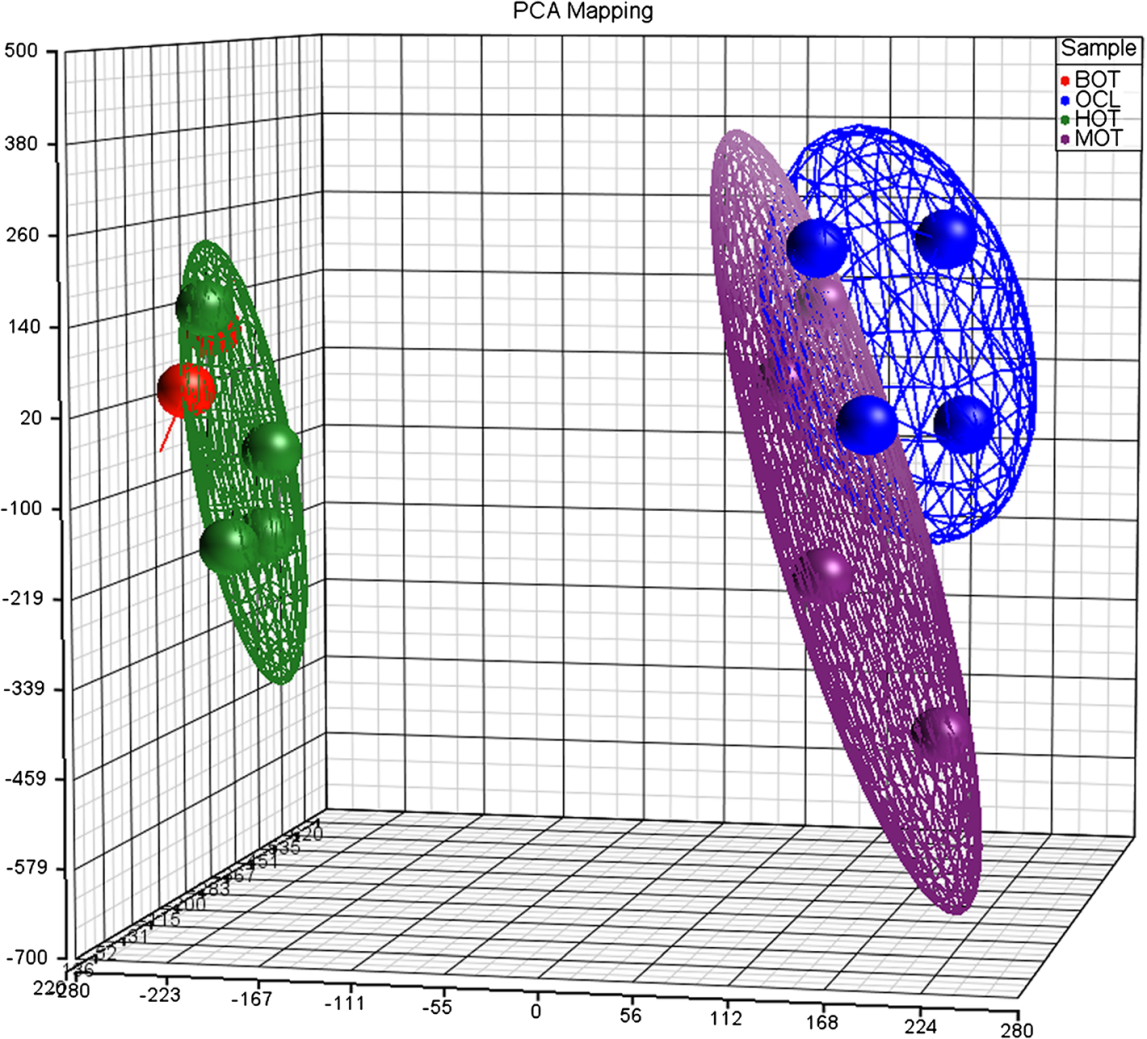
**Overlapping gene expression patterns in healthy ovary and benign tumors that differ from ovarian cancer.** Principal component analysis (PCA) depicting gene expression profiles of healthy (HOT, green), benign ovarian tumors (BOT, red), tumor (MOT, purple) and ovarian cell lines (OCL, blue).

Background correction and normalization of microarrays reported no quality control errors (QC) and, as expected, the QC intergroup proportions were variable (Additional file 1), whereas gene expression histograms were similar in all samples. General gene expression was examined and visualized according to the histological groups by means of PCA. As expected, except for HOT and BOT that essentially overlapped, MOT and OCL clustered in distinct regions of the PCA plot (Figure 1). Thus, HOT and BOT clustered together in the negative end, whereas MOT and OCL clustered separately in the positive area. This indicates that our data set has the power to discriminate OC (both MOT and OCL) from normal tissue or benign tumors. Moreover, as expected, OC gene expression profiles show a wide dispersion, reflecting tumor heterogeneity. In contrast, HOT and BOT plotted in relatively close proximity, even at the level of individual samples that for the two BOT samples practically overlapped. Finally, as expected, HOT also had some individual sample variability, probably reflecting proportional differences of tissue contents, individual variability or variations in the estrous stage, neither of which was addressed.

**Figure 2:**
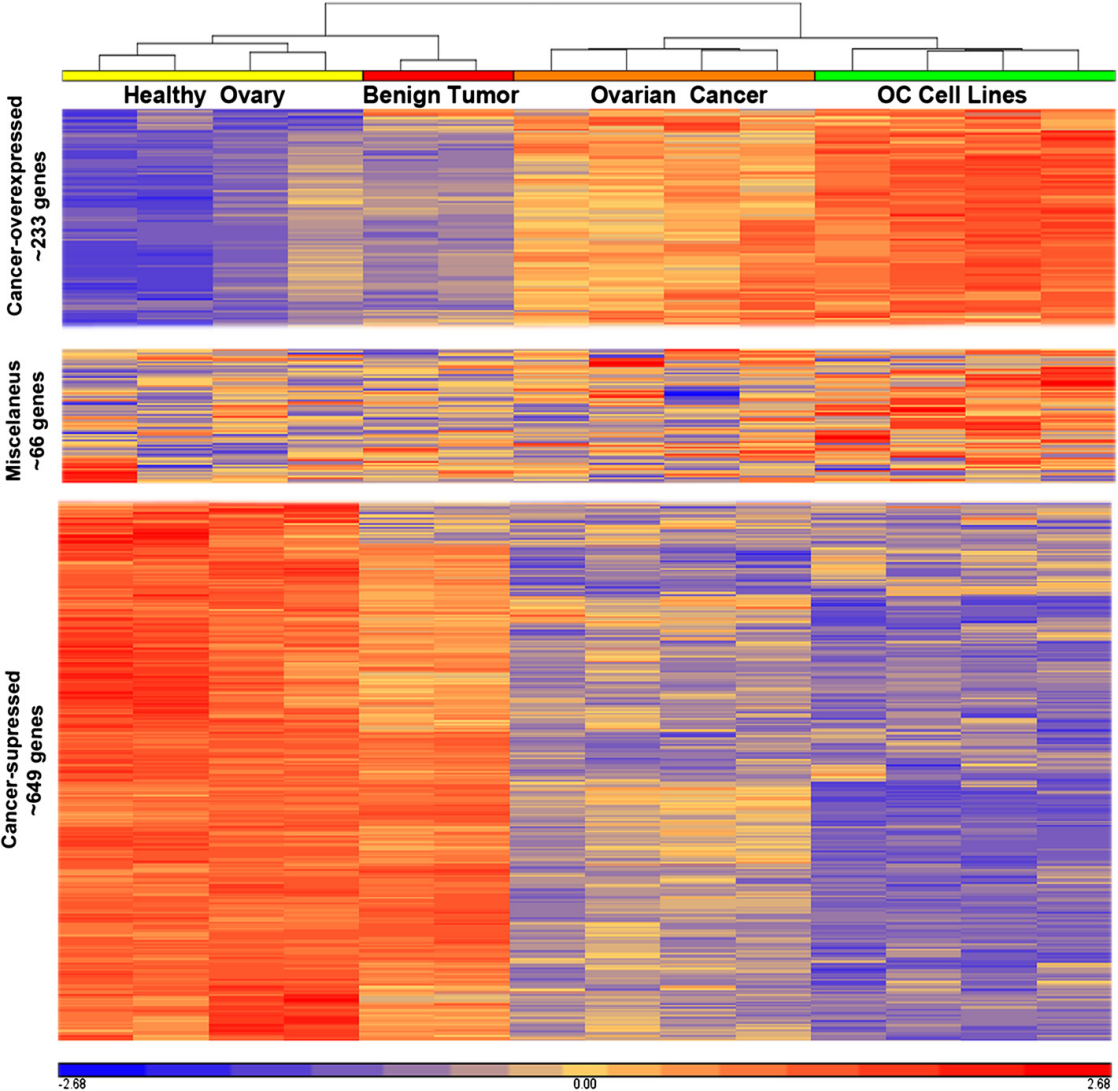
**Hierarchical clustering of ovarian cancer gene.** Heat maps depicting hierarchical clustering of gene expression in healthy ovarian tissue, benign ovarian tumours, ovarian cancer and ovarian cancer cell lines. Graphics were generated by means of Partek Genomics Suite™ software. Differentially expressed gene values were included only when fold-change was > 3 and false discovery ratio < 0.005 and only the number of genes clustering to over- expression (top), no significant changes (middle) and under-expression (bottom) are shown. Gene names, RefSeq, and other details are shown in Additional file 2.

Moreover, hierarchical clustering on the basis of relative gene expression also grouped HOT and BOT together, whereas malignancies (both MOT and OCL) clustered together but distant of HOT and BOT (Figure 2). Nonetheless, careful analysis of individual genes revealed some small intragroup differences that could reflect tumor heterogeneity and that deserves further in depth analysis. Interestingly, predominant OC-associated changes in gene expression were suppression rather than overexpression (Additional file 2).

Up to here, the results show two major ovarian tissue gene expression patterns, one specific of OC (cell lines and tumors) and the other one characteristic of borderline tumors and healthy tissue. The gross differences seen probably reflect the relatedness among the different groups. To further explore this, we compared gene expression by grouping apparently related conditions together, against each of the individual or grouped opposites (Table 1). By these means, the highest differences were MOT + OCL vs. BOT + HOT (n = 1799), followed by MOT + OCL vs. HOT (1498 differences), MOT + OCL vs. BOT (1030 differences), BOT + MOT + OCL vs. HOT (545 differences). Finally, gene expression differences between BOT and HOT were minimal (~28). On the basis of these findings, we chose to further examine the most significant differentially expressed exons.

**Table 1:** Gene expression differences among groups

**Comparisons**	**No. differences**	**Over-expressed**	**Suppressed**
BOT vs. HOT	28	24	4
MOT vs. HOT	1329	476	853
OCL vs. HOT	1664	669	995
MOT vs. BOT	625	135	490
OCL vs. BOT	1369	488	881
OCL vs. MOT	666	385	281
BOT + MOT + OCL vs. HOT	545	146	399
MOT + OCL vs. HOT	1498	508	990
MOT + OCL vs. BOT	1030	262	768
HOT + MOT + OCL vs. BOT	368	28	340
MOT + OCL vs. BOT + HOT	1799	595	1205

### Exon analysis identifies two major ovarian cancer-associated, differentially spliced transcripts of gene ZNF695

Once we had examined the relative OC-associated gene expression profiles, it was important to examine whether some of the overexpressed genes reflected only quantitative differences or if there were also qualitative differences among them. To achieve this, we performed exon analysis of genes overexpressed in both MOT and OCL. As differential exon usage cannot be easily examined in suppressed genes, we exclusively examined overexpressed genes.

**Figure 3:**
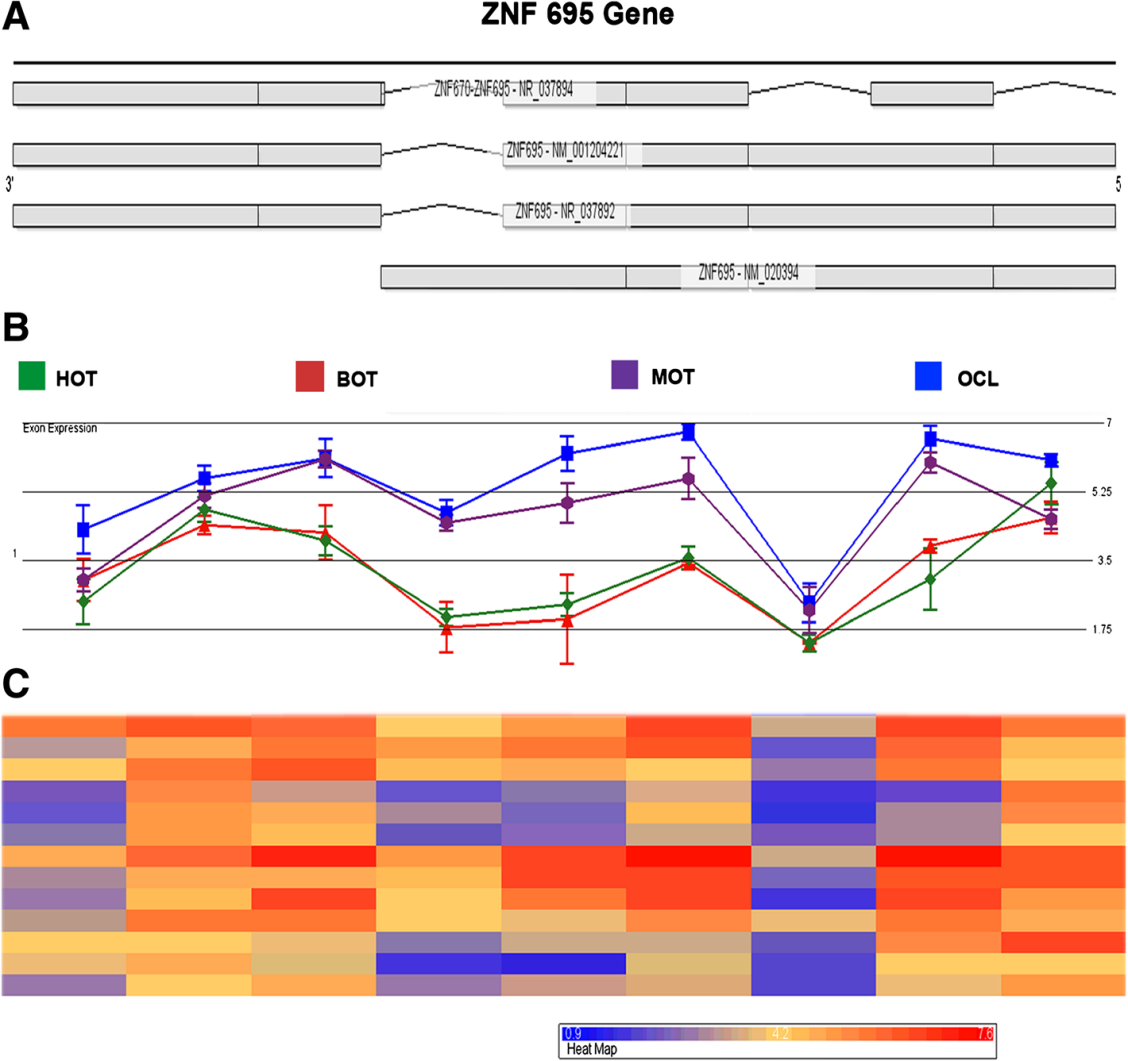
**Differential ZNF695 exon usage in ovarian cancer. A)** All possible splice variants of zinc finger protein (ZNF) 655 mRNAs retrieved from UCSC Genome Browser. **B)** Graphic depicting levels of individual exon expression in healthy ovarian tissue (HOT, green), benign ovarian tissue (BOT, red), malignant ovarian tumours (MOT, purple) and ovarian cancer cell lines (OCL, blue). **C)** Heat map of each of the peaks containing the individual samples.

The analysis was performed by means of Alternative Splicing ANOVA and the criteria to select genes for exon analysis were: false discovery ratio (FDR) < 0.05 and fold change >3, in at least one probe set. According to these criteria, the number of overexpressed alternatively spliced genes in OC was 207 (Additional file 3). To identify OC-predominant splice variants, these genes were subjected to internal analysis by comparing the expression of each individual exon for each study group against the mean total expression of the same gene in each of the groups, and were considered only when they yielded ≥3 fold change. Moreover, we performed visual inspection of each of the individual exon expression profile graphs (MOT + OCL vs. HOT + BOT or vs. HOT or vs. BOT, all of which yielded the same genes). This procedure identified at least nine genes of potential interest (Table 2), of which, ZNF695 (encoding a zinc-finger protein) had the highest overexpression (~7 fold, FDR < 0.005) in MOT and OCL, and its mRNA had the highest significant changes in exon expression with significant suppression in one its exons when compared to HOT and BOT (Figure 3). The remaining of this study focuses on the characterization of ZNF695.

**Table 2:** Main genes whit potential alternative splicing

**# of markers**	**Gene symbol**	**p-value(sample)**	**Alt-splicing(sample)**	**Fold-Change(OCL and MOT vs. BOT and HOT)**
9	ZNF695	1.28E-05	8.16E-05	4.5411
13	CCNB1	3.11E-06	2.26E-10	4.98751
36	FANCD2	4.87E-05	8.33E-10	6.58464
12	CDKN3	2.51E-04	7.59E-10	5.42259
25	TPX2	1.05E-04	2.04E-16	11.0438
14	E2F8	1.27E-03	1.92E-07	3.20517
14	FOXM1	1.09E-04	2.98E-08	5.86201
18	HMMR	1.71E-03	3.87E-02	6.55569
50	CENPE	1.87E-03	5.00E-05	4.35735

### ZNF695 splice variants in OC

ZNF695 encode a zinc finger protein with as yet unknown functions and its gene contains six exons located in chromosome 1q cytogenetic positions 247,148,625-247,171,358. This gene has six possible transcripts of which two (ZNF695-003, and ZNF695-006) encode complete ORFs yielding a 515 and a 172 amino acid length proteins, respectively; whereas the other four transcripts encode products thought to undergo nonsense-mediated decay, a process that detects nonsense mutations and prevents the expression of truncated transcripts (http://www.ensembl.org/Human/Search/Results?q=ZNF695;site=ensembl;facet_species=Human).

**Figure 4:**
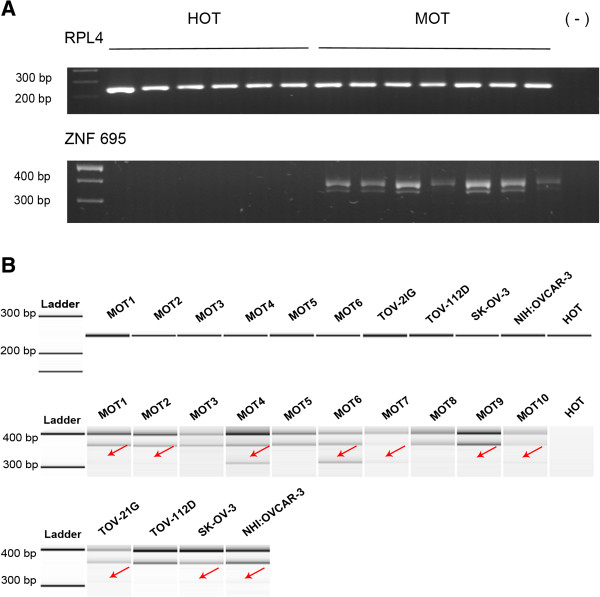
**ZNF695 transcripts of healthy ovarian tissue, individual ovarian cancer samples, and cell lines. A)** Agarose gel showing the raw RT-PCR products obtained from one HOT (n = 6) sample and MOT (n = 7) samples. Above, RPL4 protein (housekeeping), below, ZNF695. **B)** RT-PCR products run on a DNA Chip Agilent bioanalyzer. Top row: housekeeping gene RPL4. Middle and bottom rows: individual transcripts of each of the differentially spliced mRNAs in malignant ovarian cancer tissue (MOT, n = 10) and individual ovarian cancer cell lines (n = 4, shown by their names).

**Figure 5:**
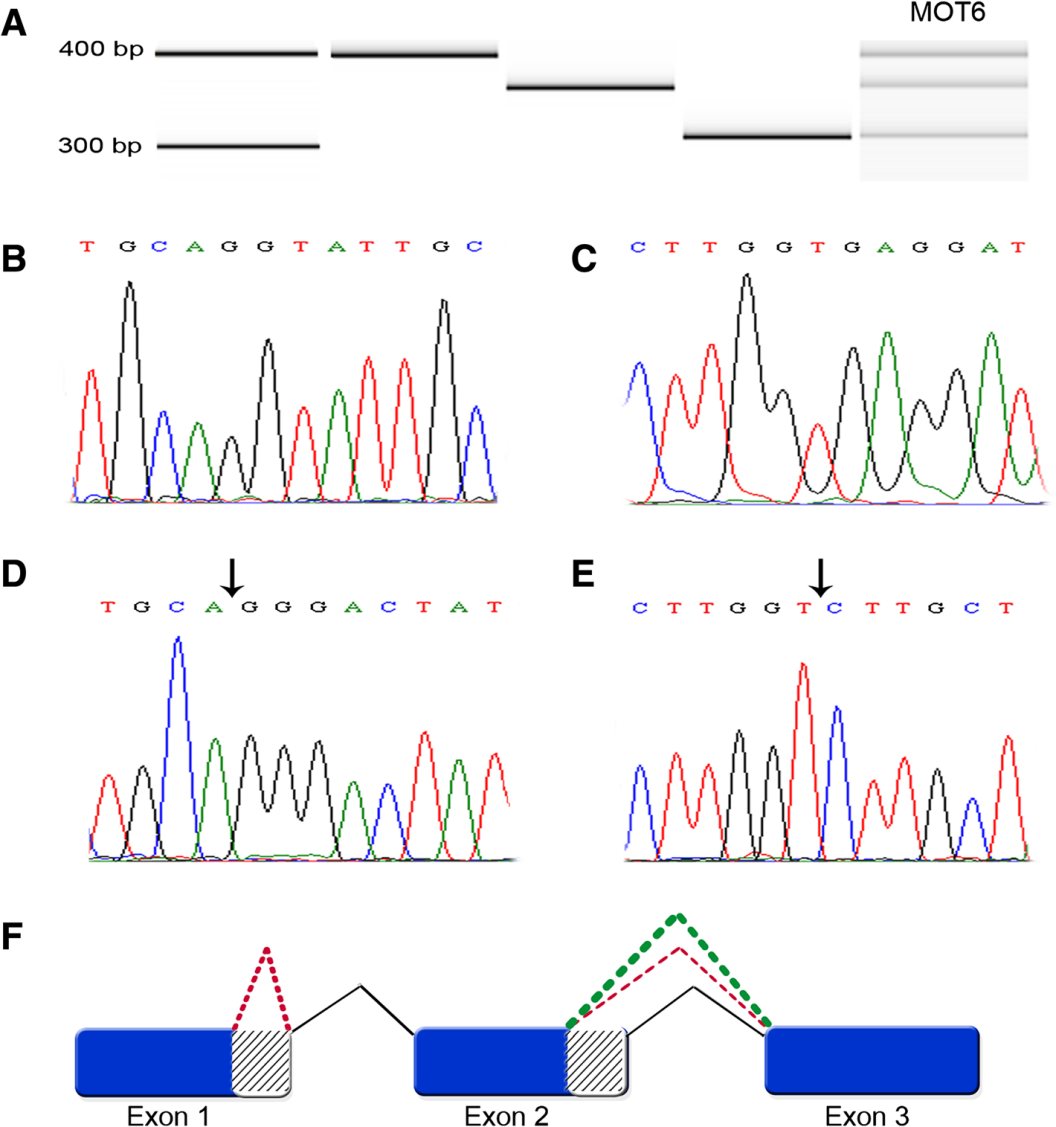
**Cloning and sequencing of novel ZNF695 transcripts associated to ovarian cancer. A)** PCR product of ZNF695 showing the three different RNA transcript sizes of ZNF695 expressed in malignant ovary tissue sample 6 (MOT6, right) and the individual amplicons used for cloning, with the size (in base pairs) shown on the left. Ladder (line 1), ZNF695 splice variants 1 and/or 2 (lane 2, 400 bp), ZNF695 splice variant 4 (lane 3, 360 bp), ZNF695 splice variant 5 (lane 4, 310 bp), and ZNF695 amplicons in MOT6 (line 5). **B)** Sanger sequence plot of ZNF695 splice variant 1 and 2 at the boundary of exons 1 and 2. **C)** Sanger sequence plot of ZNF695 splice variant 1 and 2 at the boundary of exons 2 and 3. **D)** Sanger sequence plot of ZNF695 splice 5. This transcript has alternative splicing in exon 1–2, with the arrow illustrating the alternative splicing site at the exon 1–2 boundary. **E)** Sanger sequence plot of ZNF695 splice variant 4. This sequence has alternative site splicing in exon 2–3 (arrows), this sequence are contain in ZNF695 variant 5 too. **F)** AS model of ZNF695 gene. Black lines represent primary splicing of exons 1-2-3 of ZNF695 gene containing the full coding sequence that corresponds to the largest amplicon. Green dotted line shows exons 2–3 AS, which corresponds to the intermediate amplicon. Red dotted line shows exon 1 AS together with exons 2–3 AS, which are contained in the smaller amplicon.

To characterize transcripts expressed predominantly in OC, we designed primers to identify and clone splice variants of ZNF695 most likely corresponding to the message lengths preferentially expressed in the four initial OC samples. By means of RT-PCR, in a total of 14 OC tissues (10 MOT and four OCL), expression of the three different transcripts was as follows: seven out of 10 tumor samples and three out of four cell lines expressed all three transcripts at variable degree, whereas the remaining samples (three tumors and one cell line) only expressed the two larger transcripts (Figure 4A and B). All three amplicons were gel purified, cloned into pGEM-T Easy Vector (Figure 5A) and confirmed to correspond to the aforementioned ZNF695 RNA transcripts by sequencing (Figure 5B-E). The first one appears to correspond to the full-length product (FLP), as it yields a 400 bp amplicon spanning FLP nucleotides 38 to 433 with full identity to ZNF695-003, ZNF695-006 and no other possible match (Figure 5B-C, Additional file 4). The second transcript spans 360 bp (that we name here ZNF695 transcript 4, see below) fully matching ENSEMBL nonsense-mediated decay transcript ZNF695-002, as well as NCBI peptide BAG54313.1 (Isogai, T. Helix Research Institute, Genomics Laboratory; e-mail: flj-cdna@nifty.com, http://www.ncbi.nlm.nih.gov/protein/193785160?from=1&to=118) (Figure 5B, E, Additional file 5). The third transcript (named here ZNF695 transcript 5) found here aligns with both those transcripts up to nucleotide 361, but our primers cannot identify further on the 5’ direction. Although we cannot tell to which of ZNF695 splice variants it corresponds, this transcript contains a sequence partly identical to ZFN695-002 (ZNF695 transcript 4), except that it is missing a 52 bp fragment containing part of the 5’ untranslated region and misses the translation initiation signal (Figure 5D, E, Additional file 4). Although there are additional AUG codons as potential alternative translation initiation signals 3’ of the canonical AUG, these fail to yield useful ORFs. Therefore, most likely, this transcript represents a long non-coding mRNA. Finally, Figure 5F shows the ZNF695 AS model according to the transcripts found in MOT and OCL.

## Discussion

Understanding the origin of malignancy is one of the greater challenges of modern science. Among malignant tumors, OC represents a major problem because little is known about its pathogenesis, which is also difficult to identify in early stages as it goes asymptomatic over long periods of time to be detectable only in late stages, almost always beyond any possibility of remission [33,34]. Alternative exon splicing is a biological process of major importance, because gene changes leading to altered splicing can affect normal cell and tissue function [19,20,27,35], including malignant transformation [36]. The current studies were carried out to examine whether OC could be associated to particular exon-splicing state and if so, to identify differentially spliced transcripts present in OC but absent in healthy ovarian tissue. With the exon array data set presented here, we identified nine overexpressed genes with differential exon profiles associated to OC, one such gene, ZNF695, coding for a largely uncharacterized zinc finger protein, is the most representative, with three transcripts differentially expressed by MOT and OCL, one corresponding to the whole protein, a second ORF corresponding to a shorter peptide and a third, with lower but significant expression that corresponds to a long non-coding mRNA. These results likely provide a useful biomarker of malignant transformation in women suspected to have OC and open the study of the role of these transcripts in cell proliferation and malignant transformation.

Alternative splicing is a major source of protein diversity, bioinformatics-based methods indicate that >90% human genes could be subject to AS [10,12,13] with an estimate of several million different proteins, and some individual proteins having over 1000 variants due only to AS [37]. This process can differ during distinct cellular functional or developmental stages [38,39]. It is, therefore, not surprising that AS has also been found altered during malignant transformation [40], which could be either a general marker of cancer or limited to certain cancer types. Moreover, cancer-associated AS could be the clue to understand the basis of malignant transformation, tumor behavior [23,41], and even for the identification of potential therapeutic targets [42].

We found that OC tissue has indeed a signature of alternatively spliced genes. Although we do not know yet whether these changes are indeed related only to OC or they are general markers of cancer. Of the >270 differentially spliced genes found in OC, nine were highly significant, but we decided to focus on the most significantly expressed gene with differential AS, the zinc finger protein ZNF695.

The zinc finger protein family (ZNF) spans over 700 members with many functional roles within the cell, including regulation of gene expression, which is achieved by different means. For instance, ZNF transcription factors bind to DNA by means of C_2_H_2_ zinc finger domains, constituting a subfamily of ZNF [43]. Although some ZNF members act only as repressors, others solely as act as activators, most of them can apparently be either repressors or activators depending on the particular status of the cell. Moreover, some ZNF play roles in signal transduction and many other cellular functions. ZNF695, which we found here to be differentially expressed and spliced in OC belongs to the C_2_H_2_ subfamily of ZNF and also contains Krüpple-associated box (KRAB) domains, which characteristically identify gene repressors [44-46]. Because these genes, including ZNF695 contain two or more functional domains, changes affecting only one domain can have dramatic consequences [45,47]. On one hand, repressors could lose their regulatory function or even turn in the opposite direction and become activators [47]. KRAB domains in ZNF proteins serve to bind co-repressors, which in turn mediate transcription repression [44]. Of the ZNF695 splice variants we found here to be preferentially associated to OC, isoforms 2 and 3 have incomplete KRAB domains that are essential for interactions with co-repressors which suggests that such AS pattern could be related to carcinogenesis. The third variant lacked the initial translation codon; hence, it is unlikely to yield a translational product.

Unfortunately, as yet, almost nothing is known about ZNF695 in humans or in other species, with the closest homologues having up to 64% identity. Therefore, at present it is not possible to predict how ZNF695 could play a role in OC development, if at all. One could envision that because the alternative forms found in OC have incomplete KRAB domains, this potential repressor could function as an activator and turn on cell proliferation and, hence, malignant transformation. The other possibility would be to function as dominant negative variants, but this seems unlikely because normal ovarian tissue does not express ZNF695 in any of its isoforms ad OC cells express only the alternative splice variants. Therefore, we consider ZNF695 splice variants 1/2, 4 and 5 as potential oncogenes playing a role in the pathogenesis of OC.

## Abbreviations

OC: Ovarian cancer; AS: Alternative splicing; HOT: Health ovarian tissue; BOT: Borderline ovarian tumor; MOT: Malignant ovarian tumor; OCL: Ovarian cell lines; PCA: Principal component analysis; QC: Quality control; FDR: False discovery ratio; FLP: Full-length product; KRAB: Krüpple-associated box.

## Competing interests

The authors declare that they have no competing interests.

## Authors’ contributions

SJ-M participated in the study design, carried out experiments, performed statistical analysis and helped to draft the manuscript, VV-R and RL-R performed experimental procedures, OAP-G, and MS participated in microarray analysis, VEMO-F, AZ-D participated in sequencing and cloning experiments, ML-O, RB, provided clinical sample material, performed the pathological analysis and tumor classification. JM participated in the design of the study, coordination and helped to draft the manuscript, GV-O conceived the study and participated in its design and helped to draft the manuscript. All authors read and approved the final manuscript.

## Supplementary Material

Additional file 1 **Graphic showing the proportions of four microarray quality controls.** The Y-axis depicts the expression level of controls (Log2) and the X- axis contains the 14 samples used for these studies. A) Microarray hybridization controls where the purple line corresponds to the control (CreX), green (BioDn), blue (BioC) red (BioB). B) Microaray labelling controls: pink line (dab), brown (thr), blue (phe), and orange controls (lys). Click here for file

Additional file 2 Table of genes expressed in ovarian samples. Click here for file

Additional file 3 Table of significantly over-expressed genes with potential alternative splicing. Click here for file

Additional file 4 **Alignment of ZNF695 splice variants.** BLAST analysis of sequences obtained from the clones, alternatively spliced ZNF695 variants found predominantly in malignant ovarian tissue and cell lines. Transcript variant 1/2, the sequence corresponds to the heavier band. ZNF695 Transcript variant 4, the sequence corresponds to the medium sized band, and ZNF695 splice variant 5, this sequence corresponds to the lighter band. Click here for file

Additional file 5 **Alignment of predicted ZNF695 peptides.** In silico translation of ZNF695 amplicons (ZNF695 transcript variant_1/2, ZNF695 transcript variant 4, ZNF695 transcript variant 5) revealing sequence identity of unnamed protein product (BAG54313.1) with ZNF695 transcript variants 4 and 5. ZNF695 transcript variant 1/2 is identical to full length ZNF695 (transcript variant 1 or 2). Click here for file

## References

[B1] Bast RC Jr Hennessy B Mills GB The biology of ovarian cancer: new opportunities for translation Nat Rev Cancer 2009 9 415 428 10.1038/nrc2644 19461667 PMC2814299

[B2] Scully RE Classification of human ovarian tumors Env Health Perspect 1987 73 15 25 3665859 10.1289/ehp.877315 PMC1474574

[B3] Lancaster JM Dressman HK Clarke JP Sayer RA Martino MA Cragun JM Henriott AH Gray J Sutphen R Elahi A Identification of genes associated with ovarian cancer metastasis using microarray expression analysis Int J Gynecol Cancer 2006 16 1733 1745 10.1111/j.1525-1438.2006.00660.x 17009964

[B4] Maxwell GL Chandramouli GV Dainty L Litzi TJ Berchuck A Barrett JC Risinger JI Microarray analysis of endometrial carcinomas and mixed mullerian tumors reveals distinct gene expression profiles associated with different histologic types of uterine cancer Clin Cancer Res 2005 11 4056 4066 10.1158/1078-0432.CCR-04-2001 15930340

[B5] Tinker AV Boussioutas A Bowtell DD The challenges of gene expression microarrays for the study of human cancer Cancer Cell 2006 9 333 339 10.1016/j.ccr.2006.05.001 16697954

[B6] Zorn KK Bonome T Gangi L Chandramouli GV Awtrey CS Gardner GJ Barrett JC Boyd J Birrer MJ Gene expression profiles of serous, endometrioid, and clear cell subtypes of ovarian and endometrial cancer Clin Cancer Res 2005 11 6422 6430 10.1158/1078-0432.CCR-05-0508 16166416

[B7] Keita M Bachvarova M Morin C Plante M Gregoire J Renaud MC Sebastianelli A Trinh XB Bachvarov D The RUNX1 transcription factor is expressed in serous epithelial ovarian carcinoma and contributes to cell proliferation, migration and invasion Cell Cycle 2013 12 972 986 10.4161/cc.23963 23442798 PMC3637356

[B8] Treeck O Schuler S Haring J Skrzypczak M Lattrich C Ortmann O icb-1 Gene counteracts growth of ovarian cancer cell lines Cancer Lett 2013 335 441 446 10.1016/j.canlet.2013.02.049 23474491

[B9] Lander ES Linton LM Birren B Nusbaum C Zody MC Baldwin J Devon K Dewar K Doyle M FitzHugh W Initial sequencing and analysis of the human genome Nature 2001 409 860 921 10.1038/35057062 11237011

[B10] Modrek B Resch A Grasso C Lee C Genome-wide detection of alternative splicing in expressed sequences of human genes Nucleic Acids Res 2001 29 2850 2859 10.1093/nar/29.13.2850 11433032 PMC55780

[B11] Graveley BR Alternative splicing: increasing diversity in the proteomic world Trends Genet 2001 17 100 107 10.1016/S0168-9525(00)02176-4 11173120

[B12] Carninci P Constructing the landscape of the mammalian transcriptome J Exp Biol 2007 210 1497 1506 10.1242/jeb.000406 17449815

[B13] Schmucker D Clemens JC Shu H Worby CA Xiao J Muda M Dixon JE Zipursky SL Drosophila Dscam is an axon guidance receptor exhibiting extraordinary molecular diversity Cell 2000 101 671 684 10.1016/S0092-8674(00)80878-8 10892653

[B14] Venables JP Unbalanced alternative splicing and its significance in cancer Bioessays 2006 28 378 386 10.1002/bies.20390 16547952

[B15] Hayes GM Carrigan PE Miller LJ Serine-arginine protein kinase 1 overexpression is associated with tumorigenic imbalance in mitogen-activated protein kinase pathways in breast, colonic, and pancreatic carcinomas Cancer Res 2007 67 2072 2080 10.1158/0008-5472.CAN-06-2969 17332336

[B16] Akgul C Moulding DA Edwards SW Alternative splicing of Bcl-2-related genes: functional consequences and potential therapeutic applications Cell Mol Life Sci 2004 61 2189 2199 15338051 10.1007/s00018-004-4001-7 PMC11138917

[B17] Chen LL Sabripour M Wu EF Prieto VG Fuller GN Frazier ML A mutation-created novel intra-exonic pre-mRNA splice site causes constitutive activation of KIT in human gastrointestinal stromal tumors Oncogene 2005 24 4271 4280 10.1038/sj.onc.1208587 15824741

[B18] Wang XQ Luk JM Leung PP Wong BW Stanbridge EJ Fan ST Alternative mRNA splicing of liver intestine-cadherin in hepatocellular carcinoma Clin Cancer Res 2005 11 483 489 15701831

[B19] Narla G Difeo A Reeves HL Schaid DJ Hirshfeld J Hod E Katz A Isaacs WB Hebbring S Komiya A A germline DNA polymorphism enhances alternative splicing of the KLF6 tumor suppressor gene and is associated with increased prostate cancer risk Cancer Res 2005 65 1213 1222 10.1158/0008-5472.CAN-04-4249 15735005

[B20] Thorsen K Sorensen KD Brems-Eskildsen AS Modin C Gaustadnes M Hein AM Kruhoffer M Laurberg S Borre M Wang K Alternative splicing in colon, bladder, and prostate cancer identified by exon array analysis Mol Cell Proteomics 2008 7 1214 1224 10.1074/mcp.M700590-MCP200 18353764

[B21] Adamia S Reiman T Crainie M Mant MJ Belch AR Pilarski LM Intronic splicing of hyaluronan synthase 1 (HAS1): a biologically relevant indicator of poor outcome in multiple myeloma Blood 2005 105 4836 4844 10.1182/blood-2004-10-3825 15731173 PMC1894997

[B22] Mazoyer S Puget N Perrin-Vidoz L Lynch HT Serova-Sinilnikova OM Lenoir GM A BRCA1 nonsense mutation causes exon skipping Am J Hum Genet 1998 62 713 715 10.1086/301768 9497265 PMC1376962

[B23] Singh A Karnoub AE Palmby TR Lengyel E Sondek J Der CJ Rac1b, a tumor associated, constitutively active Rac1 splice variant, promotes cellular transformation Oncogene 2004 23 9369 9380 10.1038/sj.onc.1208182 15516977

[B24] Zhou YQ He C Chen YQ Wang D Wang MH Altered expression of the RON receptor tyrosine kinase in primary human colorectal adenocarcinomas: generation of different splicing RON variants and their oncogenic potential Oncogene 2003 22 186 197 10.1038/sj.onc.1206075 12527888

[B25] Bauer TW Fan F Liu W Johnson M Parikh NU Parry GC Callahan J Mazar AP Gallick GE Ellis LM Insulinlike growth factor-I-mediated migration and invasion of human colon carcinoma cells requires activation of c-Met and urokinase plasminogen activator receptor Ann Surg 2005 241 748 756 discussion 756–748 10.1097/01.sla.0000160699.59061.92 15849510 PMC1357129

[B26] Brembeck FH Rosario M Birchmeier W Balancing cell adhesion and Wnt signaling, the key role of beta-catenin Curr Opin Genet Dev 2006 16 51 59 10.1016/j.gde.2005.12.007 16377174

[B27] Cheng C Sharp PA Regulation of CD44 alternative splicing by SRm160 and its potential role in tumor cell invasion Mol Cell Biol 2006 26 362 370 10.1128/MCB.26.1.362-370.2006 16354706 PMC1317625

[B28] Wong MP Cheung N Yuen ST Leung SY Chung LP Vascular endothelial growth factor is up-regulated in the early pre-malignant stage of colorectal tumour progression Int J Cancer 1999 81 845 850 10.1002/(SICI)1097-0215(19990611)81:6<845::AID-IJC1>3.0.CO;2-5 10362127

[B29] Hofstetter G Berger A Fiegl H Slade N Zoric A Holzer B Schuster E Mobus VJ Reimer D Daxenbichler G Alternative splicing of p53 and p73: the novel p53 splice variant p53delta is an independent prognostic marker in ovarian cancer Oncogene 2010 29 1997 2004 10.1038/onc.2009.482 20101229

[B30] Hamilton TC Young RC McKoy WM Grotzinger KR Green JA Chu EW Whang-Peng J Rogan AM Green WR Ozols RF Characterization of a human ovarian carcinoma cell line (NIH: OVCAR-3) with androgen and estrogen receptors Cancer Res 1983 43 5379 5389 6604576

[B31] Fogh J Wright WC Loveless JD Absence of HeLa cell contamination in 169 cell lines derived from human tumors J Natl Cancer Inst 1977 58 209 214 833871 10.1093/jnci/58.2.209

[B32] Provencher DM Lounis H Champoux L Tetrault M Manderson EN Wang JC Eydoux P Savoie R Tonin PN Mes-Masson AM Characterization of four novel epithelial ovarian cancer cell lines In vitro Cell Dev Biol Anim 2000 36 357 361 10.1290/1071-2690(2000)036<0357:COFNEO>2.0.CO;2 10949993

[B33] Hoskins WJ Prospective on ovarian cancer: why prevent? J Cell Biochem Suppl 1995 23 189 199 8747396 10.1002/jcb.240590926

[B34] Nguyen HN Averette HE Hoskins W Sevin BU Penalver M Steren A National survey of ovarian carcinoma. VI. Critical assessment of current International Federation of Gynecology and Obstetrics staging system Cancer 1993 72 3007 3011 10.1002/1097-0142(19931115)72:10<3007::AID-CNCR2820721024>3.0.CO;2-N 8221569

[B35] Venables JP Klinck R Koh C Gervais-Bird J Bramard A Inkel L Durand M Couture S Froehlich U Lapointe E Cancer-associated regulation of alternative splicing Nat Struct Mol Biol 2009 16 670 676 10.1038/nsmb.1608 19448617

[B36] Zardi L Carnemolla B Siri A Petersen TE Paolella G Sebastio G Baralle FE Transformed human cells produce a new fibronectin isoform by preferential alternative splicing of a previously unobserved exon EMBO J 1987 6 2337 2342 2822387 10.1002/j.1460-2075.1987.tb02509.x PMC553637

[B37] Missler M Sudhof TC Neurexins: three genes and 1001 products Trends Genet 1998 14 20 26 10.1016/S0168-9525(97)01324-3 9448462

[B38] Joseph R Dou D Tsang W Neuronatin mRNA: alternatively spliced forms of a novel brain-specific mammalian developmental gene Brain Res 1995 690 92 98 10.1016/0006-8993(95)00621-V 7496812

[B39] Chen CD Kobayashi R Helfman DM Binding of hnRNP H to an exonic splicing silencer is involved in the regulation of alternative splicing of the rat beta-tropomyosin gene Genes Dev 1999 13 593 606 10.1101/gad.13.5.593 10072387 PMC316507

[B40] Merdzhanova G Gout S Keramidas M Edmond V Coll JL Brambilla C Brambilla E Gazzeri S Eymin B The transcription factor E2F1 and the SR protein SC35 control the ratio of pro-angiogenic versus antiangiogenic isoforms of vascular endothelial growth factor-A to inhibit neovascularization *in vivo* Oncogene 2010 29 5392 5403 10.1038/onc.2010.281 20639906

[B41] Guo M Liu W Serra S Asa SL Ezzat S FGFR2 isoforms support epithelial-stromal interactions in thyroid cancer progression Cancer Res 2012 72 2017 2027 10.1158/0008-5472.CAN-11-3985 22345151

[B42] Dery KJ Gusti V Gaur S Shively JE Yen Y Gaur RK Alternative splicing as a therapeutic target for human diseases Methods Mol Biol 2009 555 127 144 10.1007/978-1-60327-295-7_10 19495693 PMC3076216

[B43] Krishna SS Majumdar I Grishin NV Structural classification of zinc fingers: survey and summary Nucleic Acids Res 2003 31 532 550 10.1093/nar/gkg161 12527760 PMC140525

[B44] Friedman JR Fredericks WJ Jensen DE Speicher DW Huang XP Neilson EG Rauscher FJ 3rd KAP-1, a novel corepressor for the highly conserved KRAB repression domain Genes Dev 1996 10 2067 2078 10.1101/gad.10.16.2067 8769649

[B45] Vissing H Meyer WK Aagaard L Tommerup N Thiesen HJ Repression of transcriptional activity by heterologous KRAB domains present in zinc finger proteins FEBS Lett 1995 369 153 157 10.1016/0014-5793(95)00728-R 7649249

[B46] Pengue G Calabro V Bartoli PC Pagliuca A Lania L Repression of transcriptional activity at a distance by the evolutionarily conserved KRAB domain present in a subfamily of zinc finger proteins Nucleic Acids Res 1994 22 2908 2914 10.1093/nar/22.15.2908 8065901 PMC310254

[B47] Agata Y Matsuda E Shimizu A Two novel Kruppel-associated box-containing zinc-finger proteins, KRAZ1 and KRAZ2, repress transcription through functional interaction with the corepressor KAP-1 (TIF1beta/KRIP-1) J Biol Chem 1999 274 16412 16422 10.1074/jbc.274.23.16412 10347202

