# Supplementary figures and images for: Splice variants of zinc finger protein 695 mRNA associated to ovarian cancer
Source: J Ovarian Res. 2013 Sep 5;6:61. doi: 10.1186/1757-2215-6-61 (PMC3847372; doi:10.1186/1757-2215-6-61)

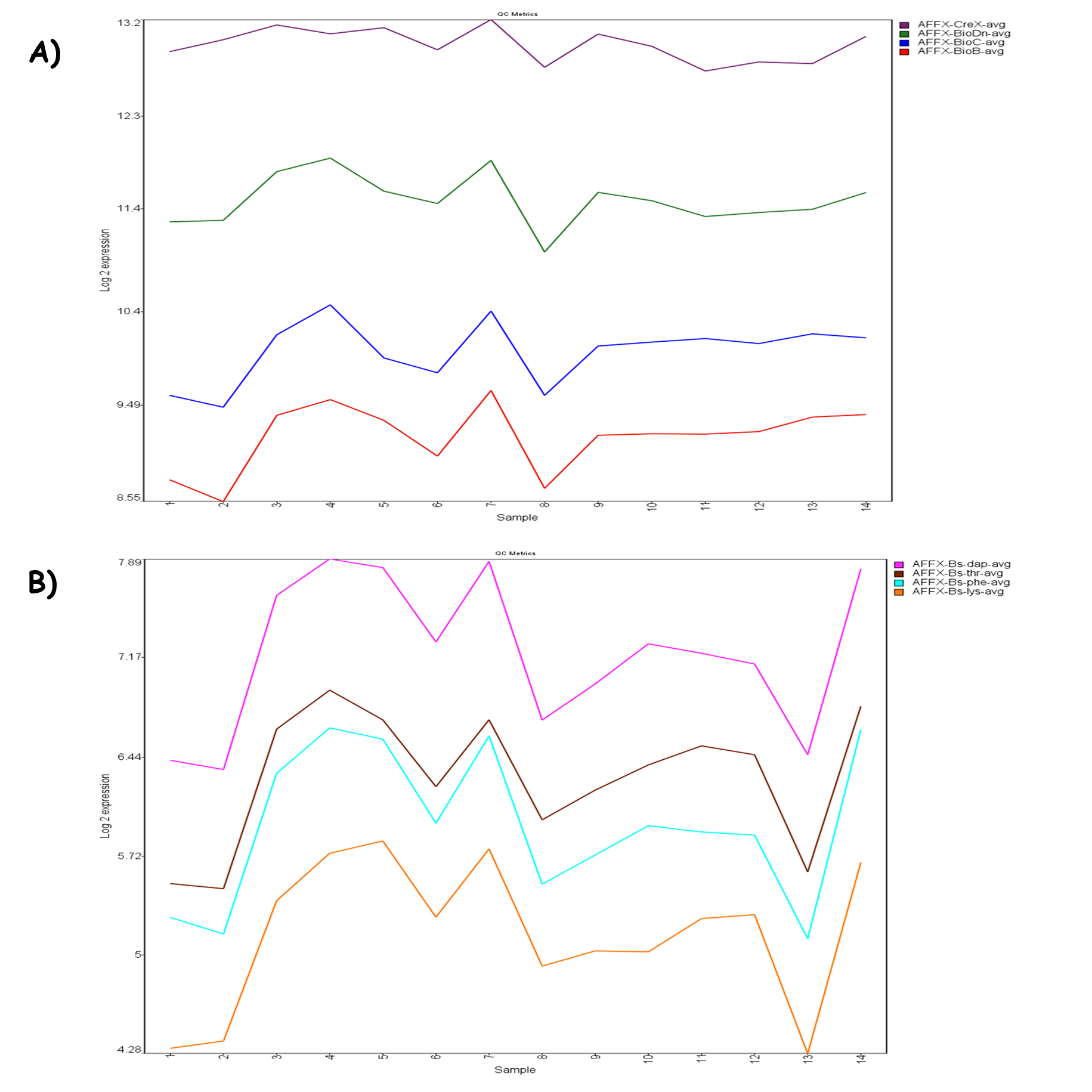

Supplement: Additional file 1 — Graphic showing the proportions of four microarray quality controls. The Y-axis depicts the expression level of controls (Log2) and the X- axis contains the 14 samples used for these studies. A) Microarray hybridization controls where the purple line corresponds to the control (CreX), green (BioDn), blue (BioC) red (BioB). B) Microaray labelling controls: pink line (dab), brown (thr), blue (phe), and orange controls (lys). [file 1757-2215-6-61-S1.tiff]
