# Supplementary material for: Splice variants of zinc finger protein 695 mRNA associated to ovarian cancer
Source: J Ovarian Res. 2013 Sep 5;6:61. doi: 10.1186/1757-2215-6-61 (PMC3847372; doi:10.1186/1757-2215-6-61)
Supplement: Additional file 4 — Alignment of ZNF695 splice variants. BLAST analysis of sequences obtained from the clones, alternatively spliced ZNF695 variants found predominantly in malignant ovarian tissue and cell lines. Transcript variant 1/2, the sequence corresponds to the heavier band. ZNF695 Transcript variant 4, the sequence corresponds to the medium sized band, and ZNF695 splice variant 5, this sequence corresponds to the lighter band. [file 1757-2215-6-61-S4.doc]

ZNF695_Full_Lenght AGAGAGGAAGGGGCGGCCTTGGGGATCTGGCGGGGCCTTTGTCTCCTTGCGGCCGGCGGG

ZNF695_Transcript_variant_1/2 --------------------------------------TTGTCTCCTTGCGGCCGGCGGG

ZNF695_Transcript_variant_4 --------------------------------------TTGTCTCCTTGCGGCCGGCGGG

ZNF695_Transcript_variant_5 --------------------------------------TTGTCTCCTTGCGGCCGGCGGG

**********************

ZNF695_Full_Lenght GTGCTGGGTTCCCGTCTGCTGCCTCTCGGAGAGTCCCGGGTGACTGCCGCAGGCTCCATC

ZNF695_Transcript_variant_1/2 GTGCTGGGTTCCCGTCTGCTGCCTCTCGGAGAGTCCCGGGTGACTGCCGCAGGCTCCATC

ZNF695_Transcript_variant_4 GTGCTGGGTTCCCGTCTGCTGCCTCTCGGAGAGTCCCGGGTGACTGCCGCAGGCTCCATC

ZNF695_Transcript_variant_5 GTGCTGGGTTCCCGTCTGCTGCCTCTCGGAGAGTCCCGGGTGACTGCCGCAGGCTCCATC

************************************************************

ZNF695_Full_Lenght GCCCTGTGGCCTGCAGGTATTGCGAGATTTATAGGGAGGACGCTGGGACCCCCAAAAGCT

ZNF695_Transcript_variant_1/2 GCCCTGTGGCCTGCAGGTATTGCGAGATTTATAGGGAGGACGCTGGGACCCCCAAAAGCT

ZNF695_Transcript_variant_4 GCCCTGTGGCCTGCAGGTATTGCGAGATTTATAGGGAGGACGCTGGGACCCCCAAAAGCT

ZNF695_Transcript_variant_5 GCCCTGTGGCCTGCA---------------------------------------------

***************

ZNF695_Full_Lenght GGGAAATGGGACTATTGGCATTCAGGGATGTGGCTCTAGAATTCTCTCCAGAGGAGTGGG

ZNF695_Transcript_variant_1/2 GGGAAATGGGACTATTGGCATTCAGGGATGTGGCTCTAGAATTCTCTCCAGAGGAGTGGG

ZNF695_Transcript_variant_4 GGGAAATGGGACTATTGGCATTCAGGGATGTGGCTCTAGAATTCTCTCCAGAGGAGTGGG

ZNF695_Transcript_variant_5 -------GGGACTATTGGCATTCAGGGATGTGGCTCTAGAATTCTCTCCAGAGGAGTGGG

*****************************************************

ZNF695_Full_Lenght AATGCCTGGACCCAGCTCAGCGGAGTTTGTATAGGGATGTGATGTTAGAGAACTACAGAA

ZNF695_Transcript_variant_1/2 AATGCCTGGACCCAGCTCAGCGGAGTTTGTATAGGGATGTGATGTTAGAGAACTACAGAA

ZNF695_Transcript_variant_4 AATGCCTGGACCCAGCTCAGCGGAGTTTGTATAGGGATGTGATGTTAGAGAACTACAGAA

ZNF695_Transcript_variant_5 AATGCCTGGACCCAGCTCAGCGGAGTTTGTATAGGGATGTGATGTTAGAGAACTACAGAA

************************************************************

ZNF695_Full_Lenght ACCTGATCTCCCTTGGTGAGGATAGCTTCAATATGCAATTCCTATTTCACAGTCTTGCTA

ZNF695_Transcript_variant_1/2 ACCTGATCTCCCTTGGTGAGGATAGCTTCAATATGCAATTCCTATTTCACAGTCTTGCTA

ZNF695_Transcript_variant_4 ACCTGATCTCCCTTGGT------------------------------------CTTGCTA

ZNF695_Transcript_variant_5 ACCTGATCTCCCTTGGT------------------------------------CTTGCTA

***************** *******

ZNF695_Full_Lenght TGTCTAAGCCAGAACTGATCATCTGTCTGGAGGCAAGGAAAGAGCCCTGGAACGTGAACA

ZNF695_Transcript_variant_1/2 TGTCTAAGCCAGAACTGATCATCTGTCTGGAGGCAAGGAAAGAGCCCTGGAACGTGAACA

ZNF695_Transcript_variant_4 TGTCTAAGCCAGAACTGATCATCTGTCTGGAGGCAAGGAAAGAGCCCTGGAACGTGAACA

ZNF695_Transcript_variant_5 TGTCTAAGCCAGAACTGATCATCTGTCTGGAGGCAAGGAAAGAGCCCTGGAACGTGAACA

************************************************************

ZNF695_Full_Lenght CAGAGAAGACAGCCAAACACTCAGTTTTGTCTTCTTATCTTACTGAAGACATTTTGCCAG

ZNF695_Transcript_variant_1/2 CAGAGAAGACAGC-----------------------------------------------

ZNF695_Transcript_variant_4 CAGAGAAGACAGC-----------------------------------------------

ZNF695_Transcript_variant_5 CAGAGAAGACAGC-----------------------------------------------

*************
