# Supplementary material for: Splice variants of zinc finger protein 695 mRNA associated to ovarian cancer
Source: J Ovarian Res. 2013 Sep 5;6:61. doi: 10.1186/1757-2215-6-61 (PMC3847372; doi:10.1186/1757-2215-6-61)
Supplement: Additional file 5 — Alignment of predicted ZNF695 peptides. In silico translation of ZNF695 amplicons (ZNF695 transcript variant_1/2, ZNF695 transcript variant 4, ZNF695 transcript variant 5) revealing sequence identity of unnamed protein product (BAG54313.1) with ZNF695 transcript variants 4 and 5. ZNF695 transcript variant 1/2 is identical to full length ZNF695 (transcript variant 1 or 2). [file 1757-2215-6-61-S5.docx]

NP_065127.4 MGLLAFRDVALEFSPEEWECLDPAQRSLYRDVMLENYRNLISLGEDSFNMQFLFHSLAMS

BAG54313.1 MGLLAFRDVALEFSPEEWECLDPAQRSLYRDVMLENYRNLISLG------------LAMS

ZNF695_Transcript_variant_1/2 MGLLAFRDVALEFSPEEWECLDPAQRSLYRDVMLENYRNLISLGEDSFNMQFLFHSLAMS

ZNF695_Transcript_variant_4 MGLLAFRDVALEFSPEEWECLDPAQRSLYRDVMLENYRNLISLG------------LAMS

ZNF695_Transcript_variant_5 -GLLAFRDVALEFSPEEWECLDPAQRSLYRDVMLENYRNLISLG------------LAMS

******************************************* ****

NP_065127.4 KPELIICLEARKEPWNVNTEKTAKH

BAG54313.1 KPELIICLEARKEPWNVNTEKTAKH

ZNF695_Transcript_variant_1/2 KPELIICLEARKEPWNVNTEKT---

ZNF695_Transcript_variant_4 KPELIICLEARKEPWNVNTEKT---

ZNF695_Transcript_variant_5 KPELIICLEARKEPWNVNTEKT---

**********************
